# Jewish Medical Students and Graduates at the Universities of Padua and Leiden: 1617–1740^*^

**DOI:** 10.5041/RMMJ.10103

**Published:** 2013-01-30

**Authors:** Kenneth Collins

**Affiliations:** Editor of Vesalius, Journal of the International Society for the History of Medicine; Research Fellow, Centre for the History of Medicine, University of Glasgow, Scotland, UK; and Visiting Professor, Department of the History of Medicine, Hebrew University of Jerusalem, Israel

**Keywords:** Jewish medical students, Jewish physicians, Padua

## Abstract

The first Jewish medical graduates at the University of Padua qualified in the fifteenth century. Indeed, Padua was the only medical school in Europe for most of the medieval period where Jewish students could study freely. Though Jewish students came to Padua from many parts of Europe the main geographical sources of its Jewish students were the Venetian lands. However, the virtual Padua monopoly on Jewish medical education came to an end during the seventeenth century as the reputation of the Dutch medical school in Leiden grew. For aspiring medieval Jewish physicians Padua was, for around three hundred years, the first, simplest, and usually the only choice.

## INTRODUCTION

The story of Jewish medical students and graduates at the Medical School of the University of Padua from the first years of the fifteenth century has been described at length.^1^–^6^ These studies have either tended to focus on specific Jewish physicians or have simply referred to the presence of Jewish students in Padua and the conditions they experienced while in Italy. Ruderman has described the encounter between Jewish students and their Christian colleagues and has pointed to Padua as the first source of “a definable social and cultural group of Jewish intellectuals.”^6^

In this paper I will show how the virtual Padua monopoly on Jewish medical education came to an end during the seventeenth century after being unchallenged for three hundred years, while the reputation of the Dutch medical school in Leiden grew. Further, through a detailed examination of graduation records, the paper will indicate that though Jewish students came to Padua from many parts of Europe the main geographical sources of Jewish students were the Venetian lands. (Modena and Morpurgo^7^ listed every Jewish graduate in Padua between 1617 and 1816.) The number of students who came to Padua from territories controlled by Venice is an indication of what might happen in other places in more tolerant times. For aspiring medieval Jewish physicians Padua was the first, simplest, and usually the only choice.

The attitude of university and ecclesiastical authorities to Jewish physicians in medieval Europe varied between self-interested acceptance and outright hostility, with an absence of consistency on every measure between the two extremes. The first universities were usually ecclesiastical institutions with close links between learning and Christian theology. The lack of access to a university medical degree did not completely restrict access for Jews to the medical profession as medical education in Europe in the Middle Ages consisted mainly of training through apprenticeship, under the guidance of an established master. The teaching experience could be completed by the conferring of a license to practice. While the general licenses issued to Jewish physicians entitled them to treat only Jewish patients, this condition was not always observed.^2^ Besides physicians, surgeons, and barbers, the medieval patient might also consult herbalists, pharmacists, and a wide variety of female healers.^8^

**Figure 1:**
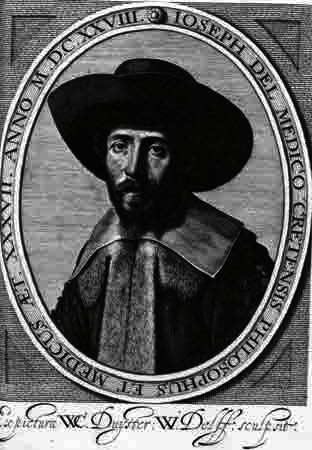
**Rabbi Joseph Solomon Qandia Delmedigo (1591–1655) was a rabbi, author, physician, mathematician, and music theorist. He was a student in Padua in 1609–1610.** From Wikipedia, http://en.wikipedia.org/wiki/File:Delmedigo.jpg, accessed January 14, 2013.

Though Jews were excluded from medieval universities, which became the norm for the training of physicians, Jews continued to aspire to the practice of medicine, and leaders of church and state often preferred to consult Jewish doctors.^9^ At the same time, Jewish community leaders worried about the risks that accompanied the exposure of their sons to university learning in the Christian world. Rabbis, like Joseph Solomon Delmedigo (Figure 1), frequently expressed their concern or even expressed their complete opposition to such studies. Rabbi Jacob David Provenzal wrote, in 1490, to Rabbi David Messer Leon his total opposition to all secular learning, including even that of medicine.^10^ Rabbi Joseph Solomon Delmedigo (1591–1655), a native of Crete and a former student in Padua, had a knowledge of logic, natural philosophy, metaphysics, and divinity and devoted himself to medicine, writing *Refu’ot Te’alah* (*Healing Medicine*), and to mathematics and astronomy. Nevertheless, in his *Sefer Elim*, he warned parents against sending their sons to Padua before “the light of the Torah has shined upon them ... in order that they not turn away from it.” Tuviya Cohen, a physician whose writings illustrate the exposure to the sciences he encountered at university, counseled that “No one (Jew) in all the lands of Italy, Poland, Germany and France should consider studying medicine without first filling his belly with the written and oral Torah and other subjects.”^11^ There were bold attempts to provide Jewish facilities for medical studies in Sicily in 1466 and in Mantua in 1564, but these proved unsuccessful.^12^,^13^ Cecil Roth considered that there was some “inconclusive evidence” that the college in Mantua did operate for a few years.^10^ Studying in Padua did give Jewish students access to the local Jewish communities, both in Padua and in Venice, where there were opportunities for Jewish students to familiarize themselves with the language and subjects required for the medical course, and which were not available to them in their own communities, in an encompassing Jewish environment. Solomon Conegliano’s private tuition was praised by Tuviya Cohen in his *Maasei Tuviya*, describing Conegliano as one of the greatest physicians and philosophers of his time. The extensive family and community networks of past Jewish graduates also provided a supportive framework for Jewish students. Indeed, more than a quarter of all Jewish graduates in Padua came from just a dozen families.

## THE UNIVERSITY OF PADUA

**Figure 2:**
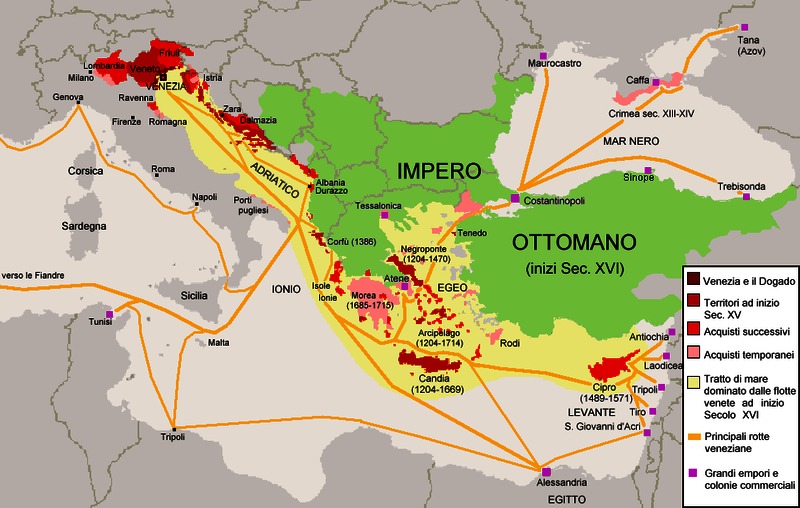
**The extent of the Venetian Empire, its commercial colonies and shipping routes.** From Wikipedia, http://en.wikipedia.org/wiki/File:Repubblica_di_Venezia.png, accessed January 14, 2013.

The University of Padua was founded in 1222, and its Medical School opened in 1250. Its status under Venetian rule from the early fifteenth century and its freedom from papal influence gave it some characteristics which did not pertain elsewhere, such as making its own policy on the admission of students. The prosperity and stability of the Venetian republic created the conditions which made it possible for Jewish students to travel across Europe to study in Padua (Figure 2). Religious divisions in Europe did not prevent Protestant or Jewish students attending this nominally Catholic university, with the first Jewish student graduating in 1409.^14^ Over the centuries it gained a reputation as a center of excellence for the quality of its teaching in its Medical School and in its other Faculties. Indeed, the Medical School was widely regarded as the best medical school in Europe. Foreign students, like William Harvey from England and many others from Britain and elsewhere in Europe, were drawn in large numbers because of the quality of the clinical teaching, rather than the formal lectures which were available in universities abroad.^15^ By the late sixteenth century students attended daily hospital rounds, and discussion of major cases, urine examination, feeling pulses, and attending autopsies were standard teaching methods.^15^

Jews had been associated with some of the earliest European universities, and while there had been occasional Jewish medical students at other Italian universities it was only in Padua where, despite regulations to the contrary, Jews managed to qualify as physicians from the early fifteenth century and on a regular and continuing basis in the subsequent centuries.^16^ While encountering petty anti-Jewish prejudices, usually in the form of fines or other financial impositions during their course of study, the opportunity offered by Padua was not equaled elsewhere in Europe before the end of the seventeenth century. Elsewhere in Italy and beyond, equal opportunities for Jewish medical students had to wait for more enlightened times. A few Jews were admitted to degrees in Siena during the seventeenth century and just a few at various times in Naples, Bologna, Rome, and Pisa, while in Livorno Jews were only admitted to medical studies in 1738.

Jewish medical students first appeared at the University of Padua in the early fifteenth century, and numbers grew gradually.^7^ Thus, while there had been only 29 graduates between 1520 and 1605, in the two centuries from 1517 there were 229 Jewish medical graduates. In the two centuries following 1617 this number grew to no less than 320, though this only averages less than two graduates each year. This increase in the number of Jewish students seems to have been associated with the transfer of authority in awarding degrees to the more secular Collegium Venetum so that by 1616 Jewish graduates regularly received the award of *doctorate in artibus et medicine* rather than the lower award of *magister*.^17^ The numbers of graduates suggest that there were probably around 10 Jewish medical students in Padua at any time during the seventeenth and eighteenth centuries, though this constituted but 1% of the total student body.^18^

An examination of the lists of Jewish physicians graduating in Padua between 1617 and 1740 (Table 1) shows the preponderance of those coming from Venetian territory. These lands include Corfu and Zante as well as Crete during the seventeenth century (Table 2). During the last decades of Venetian rule in Crete (Candia), which ended in 1669, no fewer than 10 Jews from the island managed to graduate in Padua. The presence of Ashkenazi students in Padua coming mainly from France, Germany, and Poland (Table 3) is clearly a feature of the period between 1651 and 1710 when they make up about a quarter of all the Jewish students. From this date their numbers drop substantially.

## THE UNIVERSITY OF LEIDEN

The University of Leiden, the first university in the Netherlands, was founded in 1575, and from the start its aim was to produce men of education, including physicians. Within 50 years the University attained a high status amongst European institutions of higher learning, and its medical school, led by such luminaries as Herman Boerhaave (1668–1738), probably the greatest physician of his day, eventually ensured Leiden’s enviable reputation by becoming possibly the leading European medical school during his lifetime. While there were some Jewish medical graduates from other Dutch universities, most of the graduates between 1650 and 1740, some 15 out of 25, received their degrees in Leiden.

Many of the first Jewish physicians in the Netherlands had trained in Spain, where they had been outwardly Christian and only reverted openly to Judaism once they were established in Amsterdam. From the start of Jewish communal life in the Netherlands there were regular numbers of Jews receiving licenses to practice, which could be obtained without a university education. From the middle of the seventeenth century Jews found their way into the Dutch universities, and especially into Leiden where Jewish students and graduates begin to appear around 1650.^19^ Leiden, then a town of 45,000 inhabitants, is only about 35 kilometers from Amsterdam which had already developed a significant Jewish community infrastructure. However, a Jewish community was established in Leiden, in the 1720s, by which time the Dutch medical schools had become a more popular choice for Ashkenazi Jewish students, reversing the situation between 1681 and 1710 when there had been more Ashkenazi students in Padua than in the Netherlands (Table 4).

## THE DILEMMA OF 1675

Jewish student preferences begin to change from the last quarter of the seventeenth century. By this time there was the alternative of studying in the Netherlands, and, examining the different profiles of the students in Holland and Italy, some clear differences emerge. From the first decades of the seventeenth century the proportion of students from the Venetian territories studying in Padua increases substantially. By the 1680s this proportion is more than 70% of Jewish graduates at a time when the total number of Jewish graduates in Padua begins to fall (Table 2). This pattern is emphasized when it is noted, from Modena and Morpurgo,^7^ that only four Ashkenazi students graduated at Padua between 1711 and 1740 compared to 17 between 1681 and 1710.

**Figure 3:**
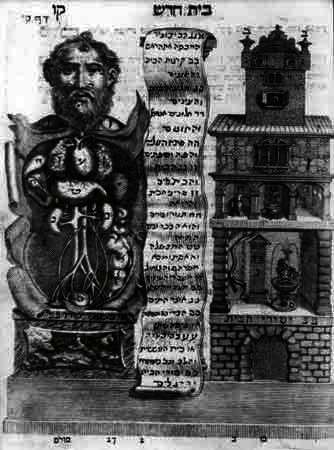
**Frontispiece of Tuviya Cohen’s *Maasei Tuviya*: the most influential Hebrew scientific and medical work of the early modern period.** From The Book of Tobias, 1708 via http://www.jewishvirtuallibrary.org/jsource/loc/loc11a.html, accessed January 14, 2013.

Tuviya Cohen is probably one of the best-known medical graduates of the Padua Medical School, through his influential and comprehensive medical and scientific work *Maasei Tuviya* published in Venice in 1707 (Figure 3), and his professional journey illustrates many of the problems faced by Jewish medical students and physicians of his times. (His name is sometimes rendered as Tobias (Toviya or Tuviya) Cohen, Cohn, Kohn, or Katz; for details of his life, times, and thought see references 20–22.) He was born in Metz in 1652 where his family had fled from Poland in 1648 during the Khmelnytsky persecutions. His father and grandfather were both rabbis and physicians, and Tuviya returned to Poland and studied at *yeshiva* in Krakow before entering the University of Frankfurt (Oder) in 1678 with a Jewish colleague, Gabriel Felix of Brody, an exceptional admission arranged through the intervention of the Great Elector of Brandenburg, Friedrich Wilhelm. However, the Great Elector could not prevent the wave of prejudice which engulfed the two students and enforced their departure from Germany. The Elector had actually arranged the admission of the two students in the hope that they might convert to Christianity. Shmuel Feiner noted^23^ that between 1678 and 1730 there were only 25 Jewish students in five universities in all of Germany.

Tuviya and Gabriel made the choice to go to Padua rather than Leiden. There was a contemporary Jewish student, Isaac Wallich, from a well-known medical family in Koblenz, who graduated in Leiden in 1675. He was not the only Jewish student there at the time, for there was a member of the Jewish community in Amsterdam who also graduated that year. Two more Jewish students finished their studies in Leiden in 1678, one a resident of Amsterdam and the other was Simon Wallich, a cousin of Isaac’s. In keeping with custom they showed evidence of previous studies, presented dissertations, and proceeded quickly to graduation.^24^

It is therefore interesting to see the name Isaac Wallich appearing again in the graduation roll of Padua in 1683, though giving Frankfurt-am-Main rather than Koblenz as his home city. We know that there *was* another Isaac Wallich studying in Halle University in 1702, receiving academic encouragement from one of Halle’s most distinguished professors, Friedrich Hoffman (1660–1742).^25^ (Wallich noted that Hoffman “tells me of all the remedies and singular secrets that he has acquired and devised ... that he will not disclose to one among thousands”. 25) Manfred Komorowski^26^ says that the two Isaac Wallichs are not to be confused (see also Modena and Morpurgo^7^), but there is no clear evidence for a third, of graduation age around 1680. If this is so, and of course there can be no proof of this as Komorowski notes, we can only conjecture that despite completing his studies in Leiden there was one Jewish student who decided to take the road to Padua for reasons which must center on the greater acceptance of the Padua degree and thus the prospects for career enhancement. Such a move by Isaac Wallich from Holland to Italy, if it happened, would be of importance in understanding the decision of Tuviya Cohen and Gabriel Felix in moving from Frankfurt (Oder) to Padua as Wallich, Cohen, and Felix all graduated from Padua in 1683.

The place of qualification of Jewish physicians practicing in the Netherlands, and almost exclusively in Amsterdam, illustrates several key differences from the graduates from the Padua Medical School (Table 5).^19^,^27^ We have noted the physicians from Spain and Portugal who reverted to Judaism in Amsterdam only after completing their medical studies in Spain or Portugal with the MD degree from such places as Salamanca, Seville, Bordeaux, and Evora. Some had found their way to Padua to study, but they arrived in Amsterdam in greater numbers where they were able to practice with their Iberian qualifications. The expulsion of the Jews from Spain had occurred in 1492, yet these Jews, who maintained their faith covertly for several generations over more than a hundred years, were still returning to an open practice of Judaism when a safe opportunity offered itself in late seventeenth century Amsterdam. There were also more than 20 Ashkenazi Jews in the Dutch lists. About a third of them had some connection with Amsterdam, whether they were born there, practiced there, or had family connections in the city. There were also about a dozen German Jews, like Isaac Wallich and his cousin Simon who graduated in 1675 and 1678, respectively.^28^ (For details of Leiden students see Molhuysen^29^ and for Dutch and German graduates Manfred Komorowski’s book.^26^)

Settled in Amsterdam, the place of study for those Jewish practitioners who wished for more than the license from the Guild of Surgeons or the Amsterdam magistrates to practice, the most popular university choices were Leiden and Utrecht. Eighty-six Jewish physicians were identified practicing in the Netherlands between 1610 and 1740. Place of study and graduation could not be identified in about a quarter of the group, and a further dozen were licensed to practice without medical degrees. Thus, about 40 Dutch Jews could be safely identified as having graduated from the local medical schools during this period, while a further 24 Jews came to universities in the Netherlands to qualify as physicians.

## CONCLUSION

In the eighteenth century the possibilities for Jews wishing to study medicine began to increase. Jews began to be admitted to the German medical schools from about 1720, and the first Jewish graduate in Scotland received his degree in 1739. Consequently the narrative of Jewish medical students changes dramatically.^30^ The story of Jewish medical students for many centuries was centered in Padua. While it attracted Jewish students from Germany and Poland, the numbers were small compared to those who were drawn from the territories under Venetian control. By the end of the seventeenth century the Dutch medical schools began to challenge this ascendancy, given their geographical proximity to the centers of Jewish population and the quality of their medical teaching and scientific development, and provided the preferred place for Ashkenazi Jewish students. This continued until opportunities grew in other European countries during the eighteenth century enabling Jewish students to study medicine in their home communities.

## Figures and Tables

**Table 1:** **Place of origin of Jewish medical graduates of the University of Padua: 1617–1740.**

**Place**	**1617–1650**	**1651–1680**	**1681–1710**	**1711–1740**
Padua	3	4	16	10
Venice	4	9	15	6
Other Northern Italy	6	6	17	10
Rest of Italy	3	1	4	12
Corfu/Zante		1	12	17
Crete	8	2		
Spain/Portugal	8			
Ashkenaz	5	14	17	4
Other/not recorded	9		4	1
Total	46	37	85	60

Data abstracted from Modena and Morpurgo.^7^

**Table 2:** **Geographical locations of the Jewish medical graduates at Padua from the Venetian Territories.**

**Date**	**Venetian**	**From Corfu or Crete**	**Total Jewish Graduates**
1617–1650	21 (45.2%)	8	46
1651–1680	22 (59.4%)	3	37
1681–1710	60 (70.6%)	12	85
1711–1740	43 (71.6%)	17	60

Data abstracted from Modena and Morpurgo.^7^

**Table 3:** **Place of origin of Ashkenazi medical students at the University of Padua.**

**Place**	**1617–1650**	**1651–1680**	**1681–1710**	**1711–1740**
Poland	2	3	9	2
Germany	3	6	6	2
France		1	1	
Other		4	1	
**Total**		14	17	4

Data abstracted from Modena and Morpurgo.^7^

**Table 4:** **Place of study of Ashkenazi medical students.**

**Place**	**1617–1650**	**1651–1680**	**1681–1710**	**1711–1740**
Netherlands	2	3	10	10
Padua	5	14	17	4
Germany				22

Data abstracted from Modena and Morpurgo,^7^ Hes,^19^ Komorowski,^26^ and Lindeboom.^27^

**Table 5:** **Place of graduation of Jewish physicians in the Netherlands: 1607–1740.**

**Place**	**Number**	**Place of Graduation**
Leiden	23	
Utrecht	10	
Other Dutch	5	
Spain	6	Salamanca, Seville
Portugal	5	Evora, Coimbra
France	3	Bordeaux, Montpellier
Licentiate (non-university	7	

Data abstracted from Hes^19^ and Lindeboom.^27^
